# Intestinal adaptations to a combination of different diets with and without endurance exercise

**DOI:** 10.1186/s12970-016-0147-6

**Published:** 2016-09-14

**Authors:** Janice L. Daniels, Richard J. Bloomer, Marie van der Merwe, Samantha L. Davis, Karyl K. Buddington, Randal K. Buddington

**Affiliations:** School of Health Studies, University of Memphis, 495 Zach Curlin Way, Memphis, TN USA

**Keywords:** Endurance training, Vegan, Gastrointestinal

## Abstract

**Background:**

Endurance athletes search for diet regimens that will improve performance and decrease gastrointestinal disturbances during training and events. Although the intestine can adapt to changes in the amount and composition of dietary inputs, the responses to the combination of endurance exercise and diet are poorly understood.

**Methods:**

We evaluated small intestinal dimensions and mucosal architecture and calculated the capacities of the entire small intestine to digest maltose and maltodextrin and absorb glucose in response to two different diet types; a western human diet and the Daniel Fast, a vegan style diet, and with moderate intensity endurance training or a no-exercise sedentary lifestyle for a 13 week period (*n* = 7 per group). The influences of diet and exercise, alone and in combination, were analyzed by analysis of variation.

**Results:**

Rats fed the western diet gained more weight (*P* < 0.05) due to more fat mass (*P* < 0.05), with a similar response for the sedentary compared with the exercised rats in each diet group (*P* < 0.05). The Daniel Fast rats had longer and heavier intestines with deeper crypts with villi that were wider (*P* < 0.05), but not taller. Despite increased energetic demands, the exercised rats had shorter and lighter intestines with shorter villi (*P* < 0.05). Yet, the percentage of mucosa did not differ among groups. Total small intestinal activities for maltase and α-glucoamylase, and capacities for glucose absorption were similar regardless of diet or exercise.

**Conclusions:**

These findings indicate the structural responses of the small intestine to a vegan style diet are modified by exercise, but without altering the capacities of the brush border membrane to digest and absorb carbohydrates.

**Electronic supplementary material:**

The online version of this article (doi:10.1186/s12970-016-0147-6) contains supplementary material, which is available to authorized users.

## Background

Sports nutrition continues to evolve from focusing on increasing glycogen stores to improving carbohydrate availability and utilization during endurance training and competition. The complex nutritional and training strategies that are used by elite marathoners [1] are founded on how the consumption of high-carbohydrate diets increase the availability and improve the oxidation of exogenous carbohydrate [2]; However, the potential involvement of intestinal adaptations are unknown.

Gastrointestinal problems are common among endurance athletes [3] and particularly for long distance runners [4–6]. The research priorities have been focused on the causes of the acute gastrointestinal disturbances that occur during training and competitive events [7] to understand how to improve the energy and fluid supplements that are consumed during activity. Less is known about the adaptive responses of the small intestine to a chronic regimen of a combination of training and dietary intake that could influence nutrient availability and the risk of gastrointestinal problems during training and competition [8, 9].

Despite early findings that pre-exercise diets high in fiber can increase the risk of GI disturbances [10], there has been interest among endurance athletes in vegan or vegetarian diets that provide adequate nutrition to fuel endurance exercise [11], are considered healthier, and improve biomarkers of cardiovascular health [12]. One vegan diet, “The Daniel Fast,” exemplifies a stringent vegan diet that allows for *ad libitum* consumption of fruits, vegetables, legumes, whole grains, nuts, and seeds and excludes all animal and processed food products, sweeteners (natural and unnatural), flavorings, preservatives, additives, alcohol and caffeine [12, 13]. The nutrient and calorie dense “Western Diet” is high in saturated fats, refined carbohydrates, sodium and cholesterol and with adverse health effects [14]. The present study examined how endurance training in combination with diets mimicking the Western diet and the Daniel Fast influences the structural and functional characteristics of the rat small intestine. The level of endurance training used for this study can be considered as light to moderate and would be relevant to individuals seeking to increase activity levels, but is much less intense than regimens typical of athletes preparing for competition.

## Methods

Male Long-Evans rats (*n* = 28, aged 3–4 weeks) were individually housed upon arrival and were allowed to acclimate for two weeks to experimental conditions, including the cage, experimental diets, handling, and protocols. All aspects of the study using animals were done in accordance with the Guide for the Care and Use of Laboratory Animals (8^th^ Edition) and were approved by the University of Memphis Animal Care and Use Committee.

During the two week acclimation period the animals were transitioned to the assigned diets by gradually replacing the standard rodent chow until at conclusion only the assigned diet was fed. The rats were placed on the treadmill on three separate days (5 min at 20 m/min) for familiarization. The 12:12 light–dark cycle was gradually shifted each day so the light phase was from 0300 to 1500.

### Dietary and exercise intervention

The rats were randomly assigned to one of four intervention groups; Western Diet with exercise (WDE; *n* = 7); Western Diet without exercise/sedentary (WDS; *n* = 7); Daniel Fast with exercise (DFE; *n* = 7); Daniel Fast without exercise/sedentary (DFS; *n* = 7). The Western Diet provided as percentages of energy 43 % carbohydrate, 40 % fat, and 17 % protein (Table 1). The Daniel Fast diet had a caloric distribution of 60 % carbohydrate, 25 % fat, and 15 % protein and was formulated with different ingredients that resulted in lower proportions of saturated fats and refined sugars (Table 2). The nutrient profiles of both diets exceeded the energy and nutrient requirements of rats, though the Daniel Fast was more similar to the 60:30:10 carbohydrate:fat:protein ratio for calories traditionally considered to be appropriate for runners [11]. The diets were formulated by Research Diets, Inc. (New Brunswick, NJ) and fed as pellets. The rats were allowed constant and unlimited access to food and water during the 13 week intervention. Body weights were recorded weekly.

**Table 1:** Macronutrient Content of the Western and Daniel Fast Diets fed to the rats

	Western Diet		Daniel Fast	
Nutrient	g/kg	kcal%	g/kg	kcal%
Protein	200	17	150	15
Carbohydrate	500	43	575	60
Fat	210^a^	40	109^b^	25
Fiber	50	0	126	0
	kcal/gm			
Energy	4.7		3.9	

^a^Percentages of fat in the Western Diet: saturated 62.4 %, monounsaturated 30.7 %, polyunsaturated 6.9 %; 2 g/kg cholesterol

^b^Percentage of fat in the Daniel Fast Diet: saturated 7.4 %, monounsaturated 18.7 %, polyunsaturated 73.9 %, no cholesterol

**Table 2:** Ingredients used to prepare the Western and Daniel Fast diets fed to the rats

Ingredient	Western Diet g/kg	Daniel Fast g/kg
Casein	195	0
Soy Protein	0	170
DL-Methionine	3	3
Corn Starch	50	0
Corn Starch-Hi Maize 260 (70 % Amylose and 30 % Amylopectin)	0	533.5
Maltodextrin 10	100	150
Sucrose	341	0
Cellulose, BW200	50	100
Inulin	0	50
Milk Fat, Anhydrous	200	0
Corn Oil	10	0
Flaxseed Oil	0	130
Vitamins, Minerals^a^	51	51

^a^(g/kg) Ethoxyquin (0.04), Mineral Mix S1001 (35), Calcium Carbonate (4), Vitamin Mix V1001 (10), Choline Carbonate (2); Ascorbic Acid Phosphate, 33 % active was added to the Daniel Fast diet (0.41)

The rats in each diet group that were assigned to endurance training of moderate intensity were placed on a moving treadmill three days per week. Following an established protocol [15], the speed and duration progressively increased from 20 m/min for 15 min/day (week 1), to 25 m/min for 30 min/day (week 2), to 25 m/min for 35 min/day (weeks 3–13). Animals assigned to the sedentary groups were placed on a stationary treadmill for 5 min three times each week to account for any potential influence of handling.

### Collection and analysis of samples

The rats were euthanized (CO_2_ inhalation) at the end of the 13 week intervention and at 72 to 96 h after the last exercise bout to avoid acute responses to exercise and thereby evaluate adaptive responses to the chronic diet and exercise regimens. The entire digestive tract was removed and placed in cold (4^0^ C) mammalian Ringers. The small intestine was isolated and after severing the mesentery, the length was measured in a relaxed state on a table top before being divided into three regions of equal length; proximal, middle, and distal. Each region was flushed with cold mammalian Ringers to remove contents, excess fluid was removed, and weight was recorded. Four segments were collected from the central portion of each region for analysis. The liver, heart, brain, and spleen were also removed and weighed.

A 3 to 4 cm segment was opened along the mesenteric border, placed on a tared piece of aluminum, and the mucosa was removed by gentle scraping with a glass slide and isolated from the underlying tissue. Total tissue mass was recorded before and after drying (60 °C for 48–72 h) and the mass of dry mucosa was recorded and used to calculate the percentage of mucosa.

Another 5 cm segment was everted and two sections were secured onto stainless steel rods to isolate 1 cm segments that were used for measuring carrier-mediated glucose uptake [16]. The tissues were suspended for 2 min in Ringers with 50 mmol D-glucose that contained tracer concentrations of ^14^C D-glucose and was aerated with 95 % oxygen and 5 % CO_2_ and mixed by a stir bar rotating at ~1,200 rpm. ^3^H L-glucose was added to the incubation solution to correct for D-glucose that was associated with the adherent fluid and passively absorbed. After the incubation the tissues were removed, placed in tared vials, weighed, solubilized (Solvable, Perkin Elmer, Waltham, MA), and scintillant was added (Ultima Gold, Perkin Elmer, Waltham, MA). Tissue accumulation of the radiotracers was measured by liquid scintillation counting (Tri-Carb 2900TR, Perkin Elmer, Waltham, MA) and calculated rates of carrier-mediated glucose uptake were normalized to tissue mass (nmol per min per mg of tissue). Rates of uptake were integrated with regional mass to estimate regional glucose uptake capacities and these values were summed to estimate the total uptake capacities of the small intestine.

Another 5 cm segment was snap frozen in liquid nitrogen and stored at −75 °C for assays of brush border membrane (BBM) maltase and α-glucoamylase activities. The frozen tissues were homogenized (Polytron; 1 g/4 ml) in 300 MHT solution (10 mM HEPES, 10 mM Trizma base, 300 mM D-mannitol, pH 7.5), CaCl_2_ was added to a final concentration of 10 mmol, the suspension was stirred for 20 min, and the cellular debris was sedimented (2,500 × g; 5 min; 4^0^ C). The supernatant was centrifuged (50,000 × g; 30 min; 4^0^ C) and the resulting BBM pellet was suspended in 400 MHT buffer (10 mM HEPES, 10 mM Trizma base, 400 mM D-mannitol, pH 7.5). The activities of maltase and α-glucoamylase were based on the amount of glucose released in 60 min at 37^0^ C [17] when the BBM were added to solutions containing 0.056 maltose and a maltodextrin with an average degree of polymerization of 5. The units of activity (1 U = 1 μmol of glucose released per minute) were normalized to protein (U/mg BBM protein; specific activity) and also integrated with regional mass to estimate total units per region and these values were summed to estimate total small intestinal BBM activities for maltase and α-glucoamylase.

The fourth segment was fixed in 10 % neutral buffered formalin, processed into paraffin, sectioned (5 μm), and stained with hematoxylin and eosin for measurement of villus height and width and crypt depth.

### Analysis of data

All data are reported as means ± SEM. One-way analysis of variance (ANOVA) was used to search for treatment effects, followed by Tukey’s post hoc test to identify specific differences among mean values. Data that were not normally distributed were tested by the nonparametric Kruskal Wallis and the Mann–Whitney U tests. *P* values < 0.05 were considered statistically significant; whereas *P* values between 0.05 and 0.10 were considered as tendencies that might become significant with larger sample sizes.

## Results

A total of 27 animals completed all aspects of this study. One rat in the WDE group died during week two of the intervention, approximately 30 min after an exercise session. The necropsy revealed the abdomen was filled with blood, with the likely cause of death a suspected aneurism. All other animals successfully completed the 13 week intervention.

### Body and organ weights

**Fig. 1:**
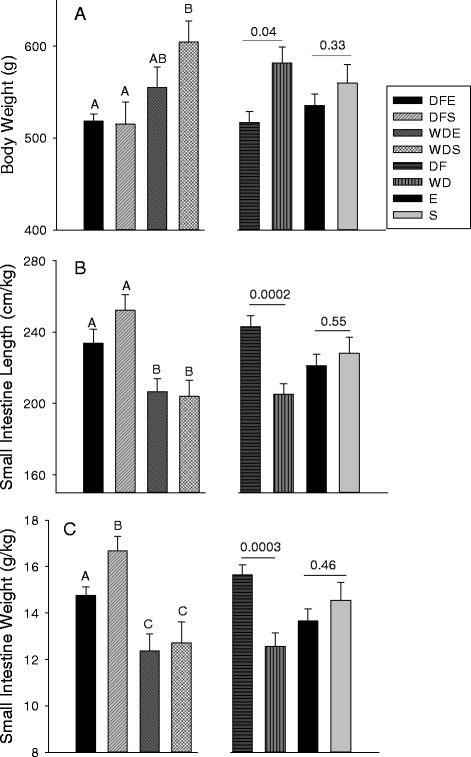
Body weights and intestinal lengths and weights normalized to body weight for rats fed the Daniel Fast (DF) and Western (WD) diets and rats either exercised (E) or sedentary (S) and for the four combinations of diet and exercise (DFE, DFS, WDE, WDS). The specific groups are represented by different bars as indicated in the panel. Individual groups with different letters are significantly different (*P* < 0.05) and *P* values for comparisons of pooled data for diets and exercise are presented

All of the surviving rats gained weight during the 13 week study (Table 3). After 13 weeks, the two groups of WD rats (pooled data for WDE and WDS) weighed more than the corresponding two groups of DF rats, and significantly so for the WDS rats (Fig. 1). Exercised rats (pooled data for DFE and WDE) were not significantly smaller than the two groups of sedentary rats (Fig. 1). The WDS rats gained the most weight, including compared with WDE rats (*P* < 0.05); whereas the DFS and DFE rats had similar weights (*P* = 0.9).

**Table 3:** Initial and final body weights (g), final body composition, and weights (g) of selected organs (means and SEM) of the rats assigned to the four treatment groups

	Daniel Fast Exercise	Daniel Fast Sedentary	Western Diet Exercise	Western diet Sedentary	P for Group Comparisons
Initial Body Mass	193 ± 3	185 ± 6	186 ± 3	187 ± 4	
Final Body Mass	519 ± 8^a^	515 ± 24^a^	555 ± 22^a^	604 ± 23^b^	<0.0001
Final Fat Mass (g)	101 ± 7^a^	124 ± 10^a^	162 ± 8^b^	195 ± 8^c^	<0.0001
Final Lean Mass (g)	391 ± 9	376 ± 8	366 ± 9	387 ± 7	
Final % Fat	20.3 ± 1.3^a^	24.6 ± 1.4^a^	30.6 ± 1.3^b^	33.5 ± 1.0^b^	<0.0001
Liver	17.2 ± 0.4^a^	16.9 ± 1.1^a^	24.5 ± 2.3^b^	27.1 ± 2.7^b^	<0.0001
Heart	1.46 ± 0.04	1.31 ± 0.08	1.42 ± 0.09	1.60 ± 0.06	
Spleen	0.75 ± 0.03	0.81 ± 0.03	0.75 ± 0.05	0.87 ± 0.03	
Brain	2.20 ± 0.04	2.12 ± 0.04	2.16 ± 0.03	2.13 ± 0.04	

Values in a row with different letter superscripts are significantly different (*P* < 0.05)

The WD rats had livers that were heavier (25.9 ± 2.0 vs 17.1 ± 0.6; *P* = 0.0002), and particularly when normalized to body weight (44 ± 1.8 vs 32.9 ± 0.5; *P* < 0.0001). Exercise did not influence liver weight, for pooled data (E = 20.2 ± 1.5 vs S = 21.6 ± 1.0; *P* = 0.60), within each diet group, and when liver weight was normalized to body weight (*P* = 0.83 for pooled data). Although absolute heart weight tended to be higher for the two groups of DF rats (1.52 ± 0.06 vs 1.38 ± 0.05; *P* = 0.09), there was no difference when heart weight was normalized to body weight (*P* = 0.44). Exercised rats did not have larger hearts when data for each diet are pooled (1.44 ± 0.05 vs 1.46 ± 0.06; *P* = 0.84) or when normalized to body weight (*P* = 0.31). However, DFE rats had hearts that were larger when normalized to body weight (1.46 ± 0.04 vs 1.31 ± 0.08; *P* = 0.03). Heart weight normalized to body weight for the WD rats did not differ between exercised and sedentary rats (*P* = 0.39). Brain weights did not differ between diet and exercise groups. Spleen mass normalized to body weight was higher for DF (1.52 ± 0.05 vs 1.40 ± 0.04; *P* = 0.05) rats and tended to be higher for sedentary (1.52 ± 0.04 vs 1.40 ± 0.05; *P* = 0.07) rats.

### Intestinal dimensions and histology

#### Responses to diet

When data for the two exercise groups (Table 4) were pooled, the intestines of DF rats were longer compared with WD rats (*P* = 0.003), with the response even more pronounced when length was normalized to body mass (Fig. 1). Although the longer intestines of DF rats did not result in the absolute mass of intestine being heavier (*P* = 0.13), the small intestines of the DF rats represented a greater proportion of body mass (Fig. 1). The higher g/kg for DF compared with WD rats extended to the three regions of small intestine (proximal: 5.8 g/kg ± 0.2 vs 4.7 ± 0.2; mid: 5.1 + 0.2 vs 4.5 ± 0.3; distal: 4.4 ± 0.2 vs 3.3 ± 0.3), significantly so for the proximal (*P* = 0.002) and distal (*P* = 0.007) regions.

**Table 4:** Intestinal dimensions and histological features (means and SEM) of the rats assigned to the four treatment groups

	Daniel Fast Exercise	Daniel Fast Sedentary	Western Diet Exercise	Western diet Sedentary	P for Group Comparisons
Intestinal Length (cm)	121 ± 4	129 ± 3	114 ± 2	122 ± 3	*P* < 0.05
Intestinal Mass (g)					
Proximal	2.85 ± 0.18	3.19 ± 0.21	2.61 ± 0.26	2.87 ± 0.15	
Mid	2.64 ± 0.17	2.65 ± 0.18	2.38 ± 0.27	2.82 ± 0.14	
Distal	2.17 ± 0.16	2.39 ± 0.21	1.87 ± 0.21	1.91 ± 0.29	
Total	7.67 ± 0.28	8.24 ± 0.55	6.87 ± 0.51	7.61 ± 0.38	*P* < 0.05
Crypt Depth					
Proximal	158 ± 5	147 ± 6	121 ± 4	125 ± 4	*P* < 0.05
Mid	137 ± 4	143 ± 5	132 ± 4	150 ± 6	*P* < 0.05
Distal	159 ± 7	143 ± 6	146 ± 5	151 ± 7	
Villus Height					
Proximal	592 ± 17	625 ± 31	512 ± 24	505 ± 24	*P* < 0.05
Mid	411 ± 23	588 ± 18	542 ± 28	563 ± 21	*P* < 0.05
Distal	320 ± 12	383 ± 11	347 ± 13	326 ± 12	*P* < 0.05
Villus Width					
Proximal	133 ± 6	152 ± 8	140 ± 5	155 ± 6	*P* < 0.05
Mid	131 ± 5	139 ± 7	157 ± 7	154 ± 7	*P* < 0.05
Distal	153 ± 7	126 ± 5	142 ± 5	131 ± 8	*P* < 0.05

The percentage of mucosa and mucosal mass did not differ between DF and WD rats in any of the three regions (data not presented). Yet, because of greater intestinal mass per kg body weight, the DF rats had more mucosal mass per kg body weight than WD rats (11.90 + 0.38 vs 10.29 + 0.67; *P* = 0.05), which was due mostly to the difference in the proximal region (4.54 g/kg ± 0.14 vs 3.76 ± 0.27; *P* = 0.01).

Differences in tissue architecture were detected among regions and diet groups (Table 4). Villus height declined from proximal to distal in DF rats; whereas the tallest villi in WD rats were measured in the middle region. As a consequence villi for DF rats were taller in the proximal region (610 μm ± 18 vs 437 ± 26; *P* < 0.0001), shorter in the middle region (502 ± 18 vs 561 ± 18; *P* = 0.02), with similar heights for DF and WD rats in the distal region (350 ± 9 vs 335 ± 8; *P* = 0.25). Villus widths for DF and WD rats were similar in the proximal and distal regions (Ps = 0.41 and 0.64, respectively), but were narrower for DF rats in the middle region (136 ± 5 vs 157 ± 6; *P* = 0.004). Crypt depth was lower in the proximal region of WD rats (114 μm ± 6 vs 152 ± 4; *P* < 0.0001), without differences in the middle and distal regions (Ps = 0.86 and 0.73).

#### Responses to exercise

When both diet groups were pooled, exercised rats had shorter intestines compared with sedentary rats (118 cm ± 3 vs 125 ± 2; *P* = 0.02). However, there was no influence of exercise when length was normalized to body mass to account for the sedentary rats being larger (Fig. 1). Similarly, the small intestines of the larger WDS rats were longer compared with WDE rats (*p* = 0.03), but not when normalized to body mass (WDS = 204 cm/kg ± 9 vs WDE 207 ± 7; *P* = 0.83). Intestinal length of DFE and DFS rats did not differ whether expressed as cm (*P* = 0.14) or normalized to body mass (DFS = 252 ± 9 vs DFE = 234 ± 8; *P* = 0.14).

Even though regional and total intestinal weights tended to be lower for exercised rats, none of the comparisons were significant. The lack of differences was even more apparent when regional and total weights were normalized to body mass (all *P* ≥ 0.35; Fig. 1). Similarly, the percentages and the mass of mucosa in the three regions and for the entire small intestine did not differ between exercised and sedentary rats (all *P* ≥ 0.48).

When all regions were pooled, exercise was associated with shorter villi (E = 466 μm ± 17 vs S = 500 ± 17; *P* = 0.02), but due solely to the differences in the middle region (577 ± 14 vs 490 ± 21; *P* = 0.004); heights were similar for E and S rats in the proximal (*P* = 0.58) and distal regions (*P* = 0.18). Crypt depths showed the same regional responses to exercise, being shallower in the middle region of E rats (134 ± 3 vs 146 ± 4; *P* = 0.01) and similar to S rats in the proximal (*P* + 0.31) and distal regions (*P* = 0.31). Villus widths for E rats were narrower in the proximal region (*P* = 0.007), similar in the middle region (*P* = 0.87), and wider in the distal region (*P* = 0.003).

#### Influence of exercise on the responses to diet

Comparisons of DFE with DFS and WDE with WDS groups were used to evaluate the combined influences of diet and exercise on small intestine characteristics. Among the DF rats, those that were exercised appeared to have shorter intestines, whether as absolute (cm; Table 4) or normalized (cm/kg), though a larger sample size is needed to verify. In contrast, exercise did not alter intestinal length among WD rats when normalized to body mass. Similarly, exercised reduced intestinal mass normalized to body mass for DF rats, but had no influence for WD rats. Within each diet group, exercise had no influence on the amount and percentage of mucosa in each region.

The responses of small intestinal histology to the combined influences of diet and exercise varied among regions. In the proximal region exercise caused crypt depth to decrease among WD (*P* < 0.001), but slightly increase among DF (*P* = 0.11) rats. Exercise did not alter villus heights of the DF and WD rats (*P* = 0.40 and 0.86), but in both groups caused a decrease in villus width (*P* = 0.07 and 0.04). The patterns of response to exercise also differed between diet groups in t the middle segment. Exercise again caused a decrease in crypt depth for WD rats (*P* = 0.01), but did not alter villus height (*P* = 0.93) or width (*P* = 0.58); whereas villus height increased in DF rats (*P* = 0.001), but crypt depth and villus width did not differ between DFE and DFS rats (*P* = 0.32 and 0.24). Histological characteristics of the distal region did not differ between WDE and WDS. In contrast, DFE rats had deeper crypts (*P* = 0.08) and villi that were taller (*P* = 0.002) and narrower (*P* = 0.02).

### Brush border membrane carbohydrases and glucose uptake

**Fig. 2:**
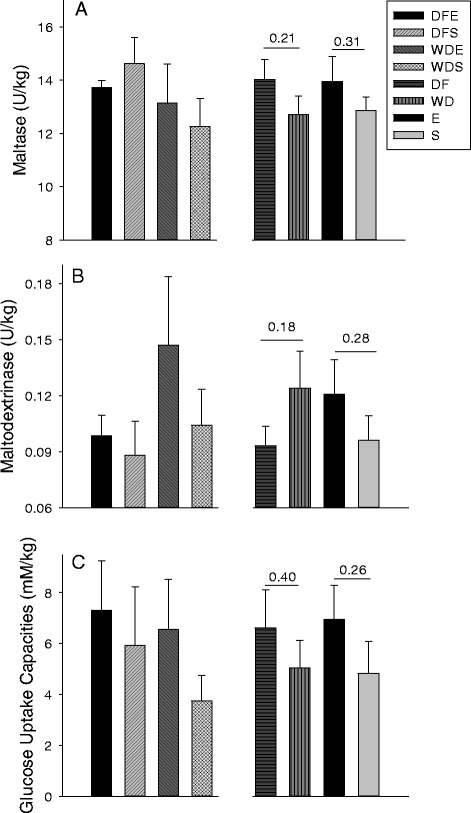
Total small intestinal maltase and α-glucoamylase activities and total small intestine capacities to absorb glucose normalized to body weight for rats fed the Daniel Fast (DF) and Western (WD) diets and rats either exercised (E) or sedentary (S) and for the four combinations of diet and exercise (DFE, DFS, WDE, WDS). The specific groups are represented by different bars as indicated in the panel. Differences were not detected as indicated by the *P* values for comparisons of pooled data for diets and exercise

Total small intestine maltase activity did not differ among groups (Fig. 2). Although the WD rats, both E and S, had higher maltase specific activity in the proximal region (0.10 U/mg BBM protein ± 0.01 U/mg BBM protein vs 0.07 ± 0.01; *P* = 0.01), total small intestinal maltase activity was not higher compared with the DF rats (*P* = 0.21; Fig. 2). Total small intestinal maltase activities did not differ between exercised and sedentary rats.

Specific activities for α-glucoamylase activity were lower than for maltase and did not differ among the four groups in any region (data not presented) or for total small intestine α-glucoamylase activities. When exercise and sedentary groups were pooled total small intestine α-glucoamylase activity was not higher for WD rats (Fig. 2) Comparisons of exercised and sedentary rats using pooled for both diet groups did not reveal an influence of exercise on specific and total small intestinal activities for α-glucoamylase.

Rates of carrier-mediated glucose uptake (nmol/mg-min) did not differ in any region among the four groups, when averaged for all three regions (*P* > 0.3), or when regional data and averages were pooled for comparisons of diets (DF vs WD; *P* > 0.4) or exercise regimens (exercise vs sedentary; *P* > 0.2). Total small intestine glucose uptake capacities were also similar for all comparisons (Fig. 2).

## Discussion

The increased weight gains of the WD compared with DF rats coincided with higher percentages of body fat, not increases in lean body mass, and less desirable plasma lipid profiles, higher concentrations of products of lipid and protein oxidation, and higher circulating concentrations of inflammatory markers (unpublished data). These findings in conjunction with the larger livers of the WD rats (present study) are consistent with the increased health risks associated with chronic consumption of the Western human diet. Importantly, even though exercise can diminish the weight gain associated with the WD, it does not resolve the adverse health risks associated with chronic consumption of the WD and resulted in a diminished performance response of the WDE rats after the 13 week period of training compared with the DFE rats (unpublished data).

The smaller spleens of the exercised rats (E = 0.75 g/kg ± 0.03 vs S = 0.84 ± 0.02; *P* = 0.07) corroborate reports of immunomodulation associated with endurance training [18, 19]. However, exercise did not result in WD animals having smaller livers or larger hearts, providing further evidence the WD blunts the benefits of endurance exercise. Brain weight was not responsive to either diet or exercise, as expected, even though circulatory and functional differences are possible [20].

Endurance athletes desire diets that provide adequate energy and nutrients, elicit positive metabolic adaptations of skeletal muscle, and don’t compromise gastrointestinal functions. The less than desirable health and performance responses to the nutrient dense WD exemplifies why many endurance athletes are interested in vegetarian style diets with ingredients that are less processed, don’t include animal products, have less saturated fats and simple sugars, and provide higher fiber content. The logical expectation is that such diets will elicit adaptive changes in the structural and functional characteristics of the small intestine that will maximize the delivery of nutrients. However, the responses of the small intestine to high fiber, vegan style diets independent of the potential influence of endurance exercise have not been extensively studied. The limited information suggests such diets will change intestinal dimensions [21], total gut transit times [22] and the microbiome [23].

Although food consumption by the rats was not measured, we recognize the amounts of food and calories consumed may have differed between rats fed the two diets. However, consumption of the same diet may not have differed between the exercised and sedentary rats [24]. Hence, the differences detected among the groups can be attributed to responses to diet, exercise, or the combination, and represent novel findings.

### Small intestine responses to diet composition

The proportionally longer and heavier small intestines of the DF rats corroborate previous findings for rats fed higher fiber diets [25] and are indicative of a trophic response to the vegan style diet. Moreover, the relatively longer and heavier small intestines of DFS compared with WDS rats along with the differences in mucosal architecture imply that DF results in more absorptive surface area per kg body weight, but without an increase in the percentage of mucosa.

Functionally, the WDS and DFS rats had the same capacities per kg body mass to hydrolyze maltose and maltodextrin and transport glucose. Even though the levels of carbohydrate in the two diets were not dissimilar (50 % vs 58 % for the WD and DF, respectively), the two diets did differ in the amounts of digestible carbohydrates. The dominant carbohydrate in the DF (Corn Starch-Hi Maize 260) is mostly resistant starch; whereas the WD has mostly simple and highly digestible carbohydrates, including the 341 g of sucrose that would induce expression of sucrase [26], which includes maltase activity, and might increase the activities of other brush border membrane carbohydrases. Although not measured, the resulting carbohydrates and glucose that would be presented to the brush border membrane from both diets may not have been markedly different.

### Small intestines responses to exercise training

The acute responses of the gastrointestinal tract to endurance training and during competitions have received attention because of the complications and dysfunctions that impact performance. Even the majority of those studies have focused on the stomach and colon because these regions are considered at higher risk of dysfunction. The present study addresses a general lack of understanding of the adaptive responses the small intestine to chronic endurance exercise, despite the importance for nutrient delivery.

Increased metabolic demands associated with pregnancy, lactation, and cold exposure elicit intestinal growth [27–30]. This led to the *a priori* prediction that endurance exercise would increase energy needs and thereby stimulate small intestine growth and increase brush border membrane functions [31]. However, exercised rats did not have larger intestines, though the proximal region did tend to have a higher percentage of mucosa (81 % vs 75; *P* = 0.06). Surprisingly, the exercised rats had shorter villi, due largely to the shorter villi in the middle region. Hence, exercise did not elicit an obvious trophic response.

From a functional perspective, the lack of differences between exercised and sedentary rats fed the same diet for brush border membrane carbohydrases and glucose uptake are consistent with similar dietary loads of substrates. Apparently, exercise at the intensity imposed on the rats does not cause intestinal adaptations that are independent of diet.

### Intestinal responses to combinations of diet composition and exercise

Improving nutrient availability and decreasing gastrointestinal dysfunctions during training and competition are of utmost interest to endurance athletes. The present findings represent some of the first insights into the adaptive responses of the small intestine to the combination of diet and training. The contrasting responses of intestine length to the vegan style diet (increase) and exercise (decrease) resulted in the DFS rats having the longest small intestines; whereas exercise had no influence on the shorter intestines of WD rats. The contrasting responses to the combination of diet and exercise were even more pronounced for intestine weight normalized to body mass, again revealing a response to exercise for the DF, but the WD rats. These responses of tissue architecture to the combination of diet and exercise are novel findings. Despite the different structural responses to the combination of diet and exercise, all of the rats maintained comparable small intestinal capacities to hydrolyze maltose and maltodextrin and absorb the resulting glucose.

## Conclusions

These results demonstrate that the structural responses of the rat small intestine to chronic moderate exercise differ between two diets that are representative of the western diet and a vegan style eating regimen. Yet, the functional abilities of the intestine at the level of the brush border membrane were not affected. Hence, the intestine has the potential to reduce gut size in response to endurance exercise without compromising carbohydrate digestion and absorption of glucose. The combined responses need to be determined at higher intensities of training which is more likely to cause gastrointestinal dysfunctions [31]. Moreover, it is unknown if the adaptive responses to diet and exercise affect carbohydrate availability and the potential for gastrointestinal distress during competition.

## Additional files

Additional file 1: Spreadsheet of data for brush border membrane assasys. (XLSX 117 kb) Additional file 2: Spreadsheet of data for intestinal dimenions. (XLSX 18 kb) Additional file 3: Spreadsheet of data for rates of glucose uptakes. (XLSX 118 kb) Additional file 4: Spreadsheet of data for histology measurements. (XLSX 23 kb) Additional file 5: Spreadsheet of data for intestinal wet and dry masses. (XLSX 17 kb) Additional file 6: Spreadsheet of data for measurements collected at the time of necropsy. (XLSX 16 kb)

## Abbreviations

BBM: Brush border membrane
DF: Daniel fast diet
DFE: Daniel fast diet with exercise
DFS: Daniel fast diet and sedentary
WD: Western diet
WDE: Western diet with exercise
WDS: Western diet and sedentary

## References

[1] Stellingwerff T (2012). Case study: nutrition and training periodization in three elite marathon runners. Int J Sport Nutr ExercMetab.

[2] Cox GR, Clark SA, Cox AJ, Halson SL, Hargreaves M, Hawley JA, Jeacocke N, Snow RJ, Yeo WK, Burke LM (2010). Daily training with high carbohydrate availability increases exogenous carbohydrate oxidation during endurance cycling. J Appl Physiol.

[3] de Oliveira EP, Burini RC, Jeukendrup A (2014). Gastrointestinal complaints during exercise: prevalence, etiology, and nutritional recommendations. Sports Med.

[4] Øktedalen O, Lunde OC, Opstad PK, Aabakken L, Kvernebo K (1992). Changes in the gastrointestinal mucosa after long-distance running. Scand J Gastroenterol.

[5] Stuempfle KJ, Hoffman MD (2015). Gastrointestinal distress is common during a 161-km ultramarathon. J Sports Sci.

[6] Sullivan SN (1987). Exercise-associated symptoms in triathletes. Phys Sportsmed.

[7] Nieuwenhoven V, Brouns F, Brummer RJ (1999). The effect of physical exercise on parameters of gastrointestinal function. Neurogastroenterol Motil.

[8] de Oliveira EP, Burini RC (2009). The impact of physical exercise on the gastrointestinal tract. Curr Opin Clin Nutr Metab Care.

[9] Murray R (2006). Training the gut for competition. Curr Sports Med Rep.

[10] Brouns F, Beckers E (1993). Is the gut an athletic organ? digestion, absorption and exercise. Sports Med.

[11] Eisinger M, Plath M, Jung K, Leitzmann C (1994). Nutrient intake of endurance runners with ovo-lacto-vegetarian diet and regular western diet. Zeit Ernährungswissenschaft.

[12] Bloomer RJ, Kabir MM, Canale RE, Trepanowski JF, Marshall KE, Farney TM, Hammond KG (2010). Effect of a 21 day Daniel fast on metabolic and cardiovascular disease risk factors in men and women. Lipids Health Dis.

[13] Alleman RJ, Harvey IC, Farney TM, Bloomer RJ (2013). Both a traditional and modified Daniel fast improve the cardio-metabolic profile in men and women. Lipids Health Dis.

[14] Cordain L, Eaton SB, Sebastian A, Mann N, Lindeberg S, Watkins BA, O’Keefe JH, Brand-Miller J (2005). Origins and evolution of the western diet: health implications for the 21st century. Am J Clin Nutr.

[15] Jin H, Yang R, Li W, Lu H, Ryan AM, Ogasawara AK, Van Peborgh J, Paoni NF (2000). Effects of exercise training on cardiac function, gene expression, and apoptosis in rats. Am J Physiol-Heart Circ Physiol.

[16] Karasov WH, Diamond JM (1983). A simple method for measuring intestinal solute uptake in vitro. J Comp Physiol B.

[17] Dahlqvist A (1964). Method for assay of intestinal disaccharidases. Anal Biochem.

[18] Martínez-Carrillo BE, Jarillo-Luna RA, Campos-Rodríguez R, Valdés-Ramos R, Rivera-Aguilar V.: Effect of Diet and Exercise on the Peripheral Immune System in Young Balb/c Mice. Biomed Res Int. 2015; 458470. doi: 10.1155/2015/458470 (2015) 10.1155/2015/458470 PMC4655039 26634209

[19] Nieman DC, Konrad M, Henson DA, Kennerly K, Shanely RA, Wallner-Liebmann SJ (2012). Variance in the acute inflammatory response to prolonged cycling is linked to exercise intensity. J Interferon Cytokine Res.

[20] Wang S, Chen L, Zhang L, Huang C, Xiu Y, Wang F, Zhou C, Luo Y, Xiao Q, Tang Y (2015). Effects of long-term exercise on spatial learning, memory ability, and cortical capillaries in aged rats. Med Sci Monit.

[21] Pond WG, Varel VH, Dickson JS, Haschek WM (1989). Comparative response of swine and rats to high-fiber or high-protein diets. J Anim Sci.

[22] Patten GS, Kerr CA, Dunne RA, Shaw JM, Bird AR, Regina A, Morell MK, Lockett TJ, Molloy PL, Abeywardena MY, Topping DL, Conlon MA (2015). Resistant starch alters colonic contractility and expression of related genes in rats fed a western diet. Dig Dis Sci.

[23] Zimmer J, Lange B, Frick JS, Sauer H, Zimmermann K, Schwiertz A, Rusch K, Klosterhalfen S, Enck P (2012). A vegan or vegetarian diet substantially alters the human colonic faecal microbiota. Eur J Clin Nutr.

[24] Lee JS, Bruce CR, Spriet LL, Hawley JA (2001). Interaction of diet and training on endurance performance in rats. Exp Physiol.

[25] Zhao X, Jørgensen H, Eggum BO (1995). The influence of dietary fibre on body composition, visceral organ weight, digestibility and energy balance in rats housed in different thermal environments. Br J Nutr.

[26] Weiss SL, Lee EA, Diamond J (1998). Evolutionary matches of enzyme and transporter capacities to dietary substrate loads in the intestinal brush border. Proc Natl Acad Sci U S A.

[27] Hammond KA, Kristan DM (2000). Responses to lactation and cold exposure by deer mice (Peromyscus maniculatus). Physiol Biochem Zool.

[28] Lam MM, O’Connor TP, Diamond J (2002). Loads, capacities and safety factors of maltase and the glucose transporter SGLT1 in mouse intestinal brush border. J Physiol.

[29] Musacchia XJ, Hartner AM (1970). Intestinal absorption of glucose, and blood glucose and hematocrit in pregnant and nonpregnant hamsters. Proc Soc Exp Biol Med.

[30] Ray EC, Avissar NE, Sax HC (2002). Growth factor regulation of enterocyte nutrient transport during intestinal adaptation. Am J Surg.

[31] Riddoch C, Trinick T (1988). Gastrointestinal disturbances in marathon runners. Br J Sports Med.

